# WORKING AROUND THE CLOCK: THE EFFECT OF SHIFT WORK AND SLEEP ON DEPRESSIVE SYMPTOMS

**DOI:** 10.1093/geroni/igac059.2417

**Published:** 2022-12-20

**Authors:** Cleothia Frazier

**Affiliations:** Duke University, Durham, North Carolina, United States

## Abstract

Shift work is increasingly prevalent in a 24-hour society where there is increased demand for round the clock service. However, shift work can disrupt circadian rhythms, which can negatively impact sleep. In turn, diminished sleep is associated with poor mental health. To expand prior research that reveal the independent effects of shift work and sleep on mental health, this study focuses on the interconnection between shift work, sleep, and depressive symptoms. Guided by the Stress Process Model (SPM), I examine the association between shift work and depressive symptoms and investigate whether sleep duration, sleep quality (insomnia symptoms), and sleep latency (the time it takes to fall asleep) mediate this relationship. Data was drawn from the age 50 health module of the National Longitudinal Survey of Youth 1979 cohort. The sample consisted of noninstitutionalized adults aged 51-60 (N=5,386). Findings show that shift workers had increased odds of short sleep, insomnia symptoms, and increased sleep latency compared to non-shift workers. Moreover, shift work was associated with increased depressive symptoms. However, part of the effect of shift work on depressive symptoms was indirect, operating through sleep. Specifically, short sleep during the week and on the weekend as well as insomnia symptoms mediated the relationship between shift work and depressive symptoms. The findings suggest that while engaging in shift work can negatively affect mental health, improving sleep duration and sleep quality can be effective in reducing the harmful effects of engaging in shift work during late midlife.

